# College preparation for a medical career in the United States

**DOI:** 10.1371/journal.pone.0298203

**Published:** 2024-02-13

**Authors:** Madelyn Malvitz, Noreen Khan, Lewis B. Morgenstern

**Affiliations:** 1 University of Michigan Medical School Department of Neurology, Ann Arbor, Michigan, United States of America; 2 Center for Social Epidemiology and Population Health, University of Michigan School of Public Health, Ann Arbor, Michigan, United States of America; JSMU: Jinnah Sindh Medical University, PAKISTAN

## Abstract

**Purpose:**

A college degree is required to enter medical school in the United States. A remarkably high percentage of students entering college have pre-medical aspirations but relatively few end up as medical students. As an “applied science”, education about medicine is usually thought to be beyond the purview of a liberal arts curriculum. Students therefore receive little education about a medical career, or information about the many alternative careers in health science. Instead, they take courses for Medical College Admission Test (MCAT) preparation and medical school application prerequisites in biology, chemistry, physics, and math. These classes give them little insight into a real medical career. The current report considers this mismatch between student needs in health science and available resources in colleges across the United States.

**Methods:**

A Collective Case Series framework was used to obtain qualitative data. Key informant interviews were requested from a convenience sample of representatives from 20 colleges, with six colleges providing extensive data. Three institutions collected data specifically on students who matriculated college interested in a career as a physician.

**Results:**

At these schools, one-half to one-quarter of students who said they were interested in medicine at the beginning of college ended up not applying to medical school. At each of the six schools, we saw a wide range of generally sparse academic and professional advising involvement and a very limited number of classes that discussed concepts directly related to careers in health science.

**Conclusions:**

Looking at this data, we provide a novel conceptual model as a potential testable solution to the problem of an underexposed and unprepared student population interested in medicine. This includes a brief series of courses intended to inform students about what a career in medicine would fully entail to help foster core competencies of empathy, compassion and resilience.

## Introduction

The path to becoming a doctor includes many challenges including required college classes with good grades, and excellent performance on standardized testing. Further, successful medical school applicants often have clinical experience, research involvement and participatory volunteering. The Association of American Medical Colleges (AAMC), the on-line portal used by students to apply to medical school in the United States does not provide a list of required college classes; requirements are determined by individual medical schools. The AAMC does, however, provide a list of recommended college classes which are common to most medical school entrance requirements. These include biology, chemistry, math and physics. There are no recommendations for topics outside of science and math, such as disciplines that might relate to the humanistic side of patient interactions. The furthest AAMC goes with their guidance in holistic academic preparation is a soft recommendation for a psychology class; no public health or social work-based classes are suggested.

One study showed only 16.5% of students who were pursuing a medical career at the start of college actually completed the mandatory coursework to apply to medical school by the end of college [1]. It is unclear why many students do not follow through on their initial plans to attend medical school. Possible reasons include poor college academic performance, loss of interest in health and medicine, competing interests or lack of preparation and understanding about a career in medicine. Notably absent from most pre-med curricula are the crucial aspects of human interaction critical to a medical career [2]. Humanistic physicians hold traits of seeking connections with patients and practicing mindfulness [3]. Since a lot of a young person’s humanity is gained through college experiences, these pivotal years provide an opportunity to begin building the compassionate, humble physicians of the future. Many future physicians also garner these humanistic qualities from the lived experience, but others do not.

Some of these goals can be met through the facets of a liberal arts education, such as education in communication, humanities, social sciences, and natural sciences [4]. Beyond that, the “applied science” of medicine is usually considered beyond the scope of a college curriculum, requiring students to get their medical exposure outside of college. One study showed the importance of humanistic concepts learned through shadowing physician experiences, which included improvement in “effective communication styles, establishment of rapport, and empathy” [5]. Nonetheless, many universities are not directly affiliated with a hospital or medical school with shadowing opportunities. This gives students little opportunity to engage with physicians. Students without a close relationship with a physician are limited in hearing from informed parties about the field. Pre-medical advisors are typically not physicians and therefore have not been through the process of obtaining a medical degree.

A remarkably high percentage of students entering college indicate a desire to pursue medicine as a career [1]. Many students consider this path because of their parental influence, because they perceive high earnings in the future and because, in general, medicine is a respected field [6]. Some students benefit from having family members in the medical field who can introduce them to what it is like to be a physician. A cohort study of 60 medical students found that a large majority of them had at least one parent in the medical field [6]. Other studies have shown students with familial connections have an increased ability to partake in medical preparation experiences such as shadowing [6]. Persons of color are very underrepresented in medicine. The lack of diversity in the medical field could be further perpetuated by underrepresented minority students being less likely to have relatives in the field [7]. One study stated that members of underrepresented minorities in medicine have greater difficulty finding clinical opportunities, are met with rejection when searching them out, and therefore feel less prepared for a medical career [8].

With the large population of entering college students stating an interest in pursuing this career, it is vital that students are exposed to the correct resources about medicine to move forward. Physician training is long, arduous, and expensive. Students need to be informed not only about the realities of a medical career, but also of the many other options in healthcare and science.

A 2013 review found only 19 original studies of premedical students in the United States had been published. The authors concluded that there was remarkably little original, peer-reviewed research on the premedical experience in the United States and that more attention was needed to this critical area [9]. We did a similar review and found only an additional 13 papers on the topic in the last decade since that review was published. In the current study, we utilized a Collective Case Study framework [10] to garner information about current practices in premedical education in the United States. Collective case studies facilitate identifying the similarities and differences among observations (cases) pertaining to a topic of interest and are an important qualitative research method. Generalization and replication are possible consequences of collective case studies framework. The aims and objectives of this study were to determine how college students prepare for applying to medical school and for a career in medicine. We also aimed to determine what courses were offered to prepare the compassionate physicians of the future. We studied how a representative group of colleges approached the issue of helping students decide on a medical career. We contacted pre-health advisors at 20 undergraduate universities and conducted a series of key informant interviews to obtain this data. Themes were extracted from the interview and are presented here. Because this is such an understudied and important issue, we used the results to develop a novel conceptual model of comprehensive premedical education that may be tested in future studies. From the data collected, we consider how colleges prepare students interested in healthcare careers and suggest some additional courses that might fill the gap between student needs and currently available resources.

## Methods

Collective Case Series methodology involves sequential or serial data collection to find themes with some consistency [10]. Through a convenience sample beginning on June 6, 2022 and ending on July 21, 2022, 20 United States’ universities were contacted to request quantitative information regarding their pre-medical student body at the beginning and end of college and the resources these students had access to along the way to help make a career decision. The schools were chosen based on similarity in admissions criteria and academic rigor—with accepted American College Testing (ACT, range 0–36, U.S. average 19.5) score averages ranging from 29 to 35—and the school’s emphasis on providing a liberal arts education rather than a technical one.

Each school’s pre-health or career center advising teams were contacted through email. A first email was sent explaining our project’s goals and that we were looking for quantitative information regarding the pre-medical student body at the beginning and end of college and qualitative information on the resources these students have along the way. It was also stated that all information disclosed would not be attributed to the school when published. To schools that responded to our request, an email was sent with specific questions, including “How many students come in freshman year saying they are on a pre-medical pathway? How many students from that group apply to medical school? How many get in? For those who do not get in, what do they go on to do? How many academic pre-health advisors do you have? Are there classes the advisors recommend that are outside of the Medical College Admissions Test (MCAT) subjects (Biochemistry, Biology, Chemistry, etc.)?” Respondents answered through email or via a virtual meeting at their preference. If schools did not respond to the first email, a follow-up email was sent 10 days later to restate our request with the previous questions included.

“Pre-medical” was defined as all paths pursuing an MD or DO degree. “Pre-health” is an overarching term and was defined as those spending their career in the healthcare field, including research and clinical positions. This includes professions such as dentistry, pharmacy, nursing, physician assistants, physical therapy, physicians, etc.

No information was collected on individuals and therefore there were no human subjects. Interviews provided data about organizations (schools and colleges). Based on the Common Rule his was a not-regulated study of organizations that did not involve humans or animals as research subjects, and based on the University of Michigan institutional guidance no IRB approval was sought.

## Results

### Schools

Six of the schools responded with data, labeled Universities A through F. Four responded by saying that they did not collect or could not give any data—Universities G through J. Ten did not respond, marked Universities K through T. Background information from each school including their selectivity based on high school grade point average (GPA) accepted to their college and standardized testing, average ACT admitted to their college is provided in Table 1. Schools were categorized as “small” if their student population was between 1,000–9,999 students, “medium” if their student population was between 10,000–29,999, and “large” if their student population was between 30,000–50,000. The universities varied in location—spanning from the West Coast to the East Coast—and the acceptance rates ranged from 5–75%. All schools had opportunities in research, while only some had clinical opportunities connected to their university.

**Table 1:** Characteristics of participating universities.

School	Average ACT^1^ accepted to college	Average GPA^2^ accepted to college
**Public**		
Small size		
University P	31–35	>3.75
University S	25–30	>3.75
University J	31–35	>3.75
Medium size		
University G	25–30	>4.00
University K	31–35	>3.75
University M	31–35	>4.00
Large size		
University A	31–35	>3.75
University B	25–30	>3.75
University E	25–30	>3.75
University H	31–35	>3.75
University I	31–35	>4.00
University T	25–30	>3.75
**Private**		
Small size		
University C	31–35	>4.00
University D	31–35	>4.00
University F	31–35	>4.00
University L	31–35	>3.75
University O	31–35	>3.75
Medium size		
University N	31–35	>4.00
University R	31–35	>3.75
Large size		
University Q	31–35	>4.00

^1^ACT: The American College Testing exam is an entrance exam that assesses mastery of English, math, reading, and scientific reasoning and is used by colleges to make admission decisions. Grades range from 1 to 36 (best) and 19.5 is the average across the U.S.

^2^GPA: Grade point average is a cumulative number that represents one’s grades throughout their academic career. GPA is on a scale of 0 to 4, with above 4 indicating succeeding in advanced courses.

### Themes

From the information gathered, we identified three themes that could highlight how universities approach the opportunity to direct premedical students: data surrounding the premedical population at the start and end of college; the composition and support of the academic or professional advising committee; and the types of classes offered and recommended to its students. This information is summarized in Table 2.

**Table 2:** Themes of how each university supports their pre-medical students.

	Pre-medical population throughout pre-medical journey	Academic or professional advising committee	Types of classes offered and recommended to its students
University A	Enter Pre-medical: Around 1500 students per year show interest in pursuing a medical career. Apply to Medical School: From 2019 to 2021, around 800 of the original 1500 students apply to medical school. Matriculate into Medical School or Other: About half are accepted and officially matriculate into medical school, one-fourth will reapply with uncertain outcome), and one-fourth will not be accepted and will not continue to pursue this career.	Composition: Pre-health has two advisors and pre-medical has one additional advisor. Therefore, three people are advising 6,000 pre-medical students, with two of those individuals also advising the unknown number of pre-health students. Support: Each pre-health advisor holds appointments for 10 hours a week to discuss career goals with students. Students may meet with the pre-medical advisor only during their upperclassmen years to discuss applications to medical school.	Classes: There is a class that talks about providing high-quality care to patients and medical communication. This class is taught once a year and is limited to 50 students. Other Resources: No other known resources.
University B	Enter Pre-medical: N/A, data not collected by the school. Apply to Medical School: N/A, data not collected by the school. Matriculate into Medical School or Other: 13.1% of graduates obtain a health-related degree.	Composition: This pre-health advising team consists of four full-time individuals. Support: Support questions were not answered by this school.	Classes: There is a freshman seminar course about preparing for professional health programs and academic success. Two other courses are offered on medical topics of justice in the health care system, medical communication, and holistic treatment, which is limited to 50 students per class. Other Resources: No other known resources.
University C	Enter Pre-medical: N/A, data not collected by the school. Apply to Medical School: N/A, data not collected by the school. Matriculate into Medical School or Other: Their website shows 70–80% of their applicants are accepted into medical school, but it is not known how many reapply.	Composition: Pre-health has three advisors and pre-medical has three as well. They also have alumni in the medical field that advise students occasionally. Support: Both advising teams support students’ decisions in medicine and help with the application process. Alumni give insight on a career in medicine.	Classes: They strongly recommend psychology and sociology. There are no health science classes offered. Other Resources: No other known resources.
University D	Enter Pre-medical: N/A, data not collected by the school. Apply to Medical School: In 2019, this school stated that around 3% from their graduating class applied to medical school. Matriculate into Medical School or Other: 89% were accepted. There is no information on reapplication rate.	Composition: There are two pre-medical advisors. Support: Support questions were not answered by this school.	Classes: There is a class on providing high-quality care to patients and how medical humanities and social sciences can affect health care views. Other Resources: No other known resources.
University E	Enter Pre-medical: One-third show interest in a pre-health path. Pre-medical is not subcategorized here from pre-health. Apply to Medical School: An on-line course with information on how to apply is offered by the university for students and alumni (700 people registered). Matriculate into Medical School: Around 55% of these students will matriculate into medical school, suggesting around 400 will go to medical school and 300 will either reapply to change career paths.	Composition: Pre-medical has six advisors. Some of these individuals specialize in specific pre-medical student initiatives. Support: In the 2022 school year, this team helped 2,300 students interested in pre-health careers.	Classes: They do not have classes that directly cover larger topics of medicine. Other Resources: There is a non-graded on-line course that teaches first-year students what majors fit into the pre-medical path, where to volunteer and shadow, what a four-year academic plan may look like, and what other resources are provided by the school.
University F	Enter Pre-medical: Around 300 students report they are interested in medical school upon entrance. Half of these students will continue to show interest throughout their college career. Apply to Medical School: Around 150 students apply to medical school each year. Matriculate into Medical School: Their acceptance rate is 87%. It is unknown what happens to those not accepted.	Composition: There are three full-time and a few part-time pre-health advisors. They state the large majority they advise are pre-medical. Support: Advisors work with the pre-medical students “a few times” at the start of the process and much more often at the end to help with the medical school applications and following AAMC recommendations.	Classes: They do not have classes that directly cover larger topics of medicine. Other resources: There is an extracurricular series that discusses doctor-patient communication, health disparities, professionalism, and other health professions, as well as includes a volunteering program with the school hospital and a detailed packet on how to craft an AAMC application.

The data show tremendous premedical interest at the start of college, but this number decreases drastically by the end of college. The proportion of students that officially matriculated into medical school was even lower. The advising structures were remarkably understaffed, often with advisors providing service to thousands of students. This is seen in University A’s ratio of 3,200 total students to 2 advisors. This may result in a lack of support for student decision-making. There were very few resources for college students to learn about a career in medicine through a structured learning environment, and little help for students to investigate alternative career choices in the healthcare field.

## Discussion

While it is certainly true that a liberal arts education is part of the formula needed to prepare students to be compassionate physicians, there remains a gap in the educational process that should inform students about careers in healthcare and discusses the qualities of compassion, empathy and resilience needed to be a great physician. Because such a small percentage of college students who indicate an initial interest in medicine later attend medical school, there is also a need to guide students to other careers in health. The goal is not to increase medical student applicants; the goal is to match students with the career that suits them best and to prepare future physicians to become compassionate, empathic doctors. In the current study, the largest common factor at all universities was the lack of available coursework to expose students to a full understanding of the medical field. Although the “liberal arts” structure aims to provide an educational foundation and allow students to develop professional skills after graduation, the pivotal years of college are the time to develop the humanistic and interpersonal skills needed for a career as a physician. Additionally, more specific coursework about medicine may prove beneficial for this development. Further, if students do not get into medical school or are unsure about their career path in health, early exposure to alternative careers may be helpful. Therefore, courses supporting these skills should be accessible during the undergraduate experience. Although some universities supply a few classes with more broad topics in medicine, the small class size makes them only available to a few students.

From the current data, we suggest beginning with classes that can support the many students in need of direction. There is a class at The University of Michigan cross-listed at the School of Public Health, School of Kinesiology, and Medical School (for post-baccalaureate students) that speaks to the issues raised in this paper. "The Science of Medicine" is a class that focuses on different careers in medicine, evidence-based medicine, and doctor-patient communication. The syllabus is available as a S1 File. The course is structured into classes with two parts, with the first part containing an interactive lecture and the second part a class activity to reinforce the material on a personal level. Readings include academic articles on the “Art of Medicine” and “Inequalities in health”, and many other concepts that connect the scientific parts of medicine with interpersonal interactions, public health, and humanity. Guest speakers who are active in the healthcare field discuss many different careers in medicine, giving students a deeper and more realistic understanding of their possible future. There is a connecting focus in lectures on providing evidence-based medicine, which is a practice of medicine that includes research-backed care; and shared-decision-making, which involves empathetic, and collaborative decisions on care that include input from both the physician. During the semester, patient presentations allow individuals to tell their stories about interactions with healthcare, both positive and negative. Late in the semester, students craft short videos asking friends or family to share instructive stories about healthcare interactions. These videos are then concatenated to make a full-length movie that the class watches together. Early exposure to these foundations of care shows students how science intersects with compassion and the personal aspects of medicine to lead to successful patient interactions and outcomes. This class helps students understand if medicine is something they would like to pursue in the future, or if their prior vision of the career does not match.

From the data collected, we saw a lack of educational offerings in college to support students’ preparation for careers in health science. Without a curriculum to support this understanding, students must look beyond the scope of their university for this real-life information. Often this is seen in shadowing and mentoring experiences, which although a useful supplemental resource, are not equally available to all students like college classes are [8]. Disparities in access to mentoring and shadowing opportunities are vast [6]. The current premedical curriculum contributes to the persistent lack of diversity in the medical profession [11]. A lack of curriculum may continue to exacerbate disparities in medicine by not preparing all populations equally to solidify this career.

**Fig 1:**
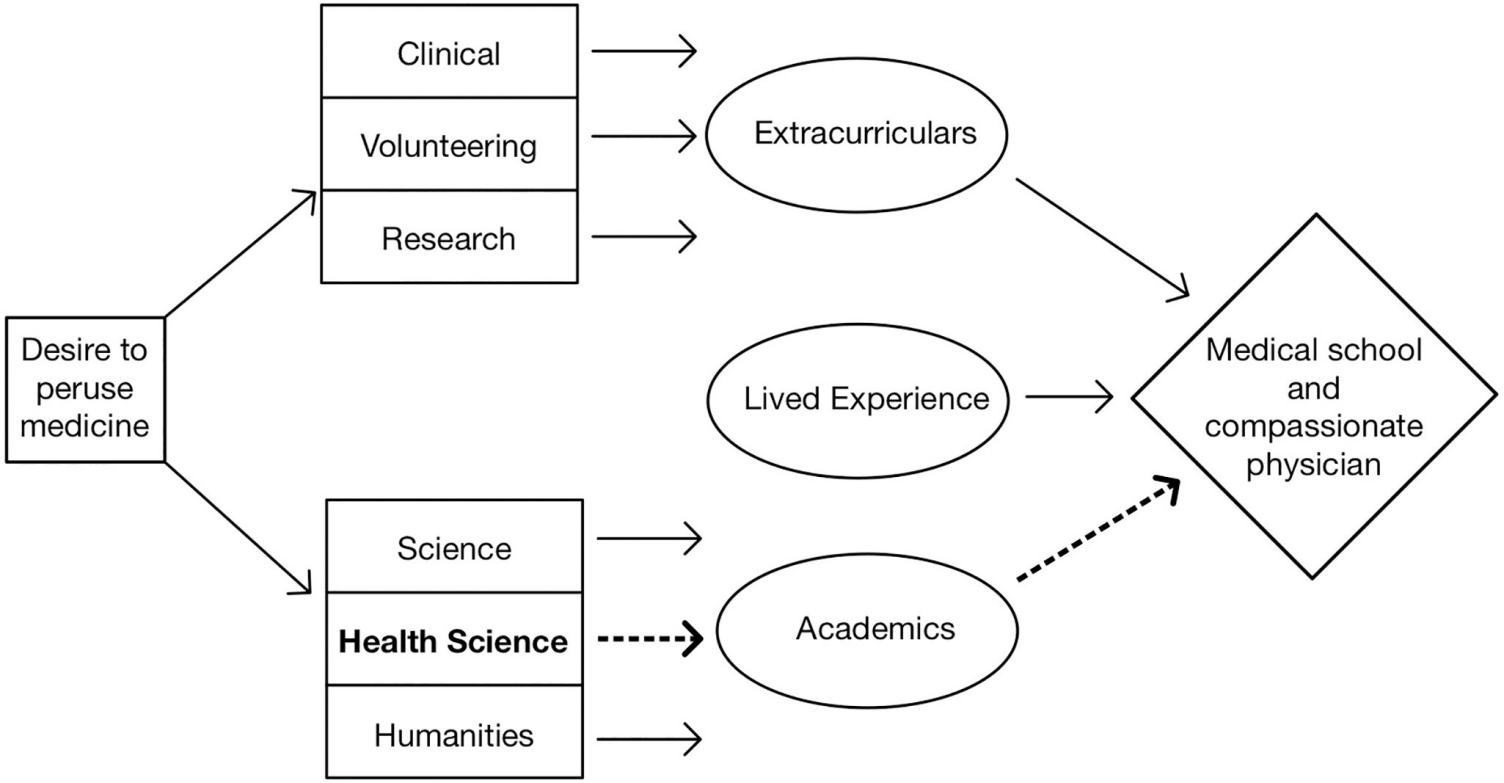
The missing link of premedical curriculum. This theoretical model suggests that core competencies for healthcare professionals including compassion, empathy and resilience would be strengthened through health science education in the pre-medical curriculum.

Based on the data presented we developed a novel conceptual model of comprehensive pre-medical education shown in Fig 1. The missing link is health science curriculum which provides more direct education about health care practice, evidence-based medicine and patient-provider shared decision making. The path from health science through academics to medical school and becoming a compassionate physician, which is essentially absent at the present, is made up of courses such as The Science of Medicine. It is there that fundamental competencies such as compassion, empathy and resilience combine with intellect and the scientific basis garnered from classwork in science. Study in the humanities as well as clinical observation (shadowing) and volunteering also promotes necessary competencies in empathy and compassion and are important to strengthen in the model. Since this model is newly proposed, these competencies and their association with available college coursework should be examined in future research. Lessons in how other disciplines prepare career professionals may also contribute to the college training of future doctors and this should be investigated.

### Limitations

Schools that did not respond to our information requests may have an academic program or professional support that was not accounted for in the discussion. It is also possible that their lack of response suggests even fewer resources than the schools that did respond. This report is based on a convenience sample of a relatively small number of schools. Larger sample sizes and a high cooperation rate would increase confidence in the data. The new theoretical model is based on the findings from this study and observations from the authors, including one professor and two medical students. It lacks contributions from the literature because there is such sparse information published in this area. Future testing and validation are needed prior to implementation of the model.

## Supporting information

S1 File Science of medicine syllabus. (DOCX)
