# Supplementary material for: College preparation for a medical career in the United States
Source: PLoS One. 2024 Feb 13;19(2):e0298203. doi: 10.1371/journal.pone.0298203 (PMC10863898; doi:10.1371/journal.pone.0298203)
Supplement: S1 File — (DOCX) [file pone.0298203.s001.docx]

**MEDPREP 470/ MOVESCI 477/ NEUROL 470/ PUBHLTH 465: The Science of Medicine**

3 Credits

Term

Date and Time

Class location:

Course Director and Instructor**:**

Class Assistant: Office hours by appointment.

Prerequisite: None although junior undergraduate status or above recommended

Course Materials:

All readings are available online and free of charge. Readings that appear in the syllabus are required and may be part of exams.

1: Ben-Shlomo Y, Brookes ST, Hickman M. Epidemiology, Evidence-based Medicine and Public Health. 6^th^ Ed. Wiley-Blackwell, 2013.

**This book is available from the Health Sciences Electronic Library:**

2: Tolstoy, Leo. The Death of Ivan Ilyich. Publisher: Bantam Books 1981.

**This book is available from the Health Sciences Electronic Library:**

Further required readings from original articles are included in the syllabus and are freely accessible to all students through the library electronic journal access. Articles from web addresses are publicly available. Articles will be available through the CANVAS course site as well. Please remember that these articles are under copyright and should not be circulated to people outside the University.

There will be additional reading provided during some lectures that are optional, for your interest only and not part of the material covered in exams.

Course Description: Everyone will interact with the health care system during their lives whether caring for family and friends, or oneself. As such, this course is valuable to all. This course provides a vital understanding for how decisions on diagnostic tests and therapies are deeply rooted in science. The scientific approach is reviewed through an appreciation of the different types of study designs in medicine and public health. The modern concept of evidence-based medicine (EBM) is highlighted throughout the course. EBM uses the scientific method to develop a rationale for diagnostic and therapeutic decision making. This is contrasted to eminence- or experienced-based medicine which relies on the individual opinions of single physicians. In the end, students will appreciate that modern medicine relies on science fundamentally, supplemented with knowledge of anatomy and physiology, and strong components of “art” formed by experience which guides the compassion, caring and personal aspects of medicine that are so crucial to good care. This course also delves deeply into the many potential careers available in the medical sciences for those considering this in their future.

Course Objectives:

Students taking this course are expected to learn about:

1. Evidence-based medicine

2. The scientific underpinnings of diagnosis and treatment

3. The ethics of medical decision making; strategies for communication, and recognizing and caring for special populations

Course Format:

The first 40 minutes of the class in general will be an interactive lecture. I invite your questions and comments during the lecture. The second 40 minutes will be a class activity that reinforces the lecture and reading material and allows you to be creative and express yourselves. Some of the class activities will be individual, but most will occur in groups. I will be available to you during the class activity part of the class to answer any questions and help direct your answers to the questions. During the last 10 minutes of the class we will reconvene to discuss the assignment together. All students in a group will post (in CANVAS Assignments) the same response to the in-class assignment with all students’ names in the group at the top. These assignments must be uploaded to CANVAS before the end of class. They are not graded, but I read them all to get a sense of how individuals and the class are mastering the material.

Students will be assigned readings with the expectation that they are completed before the class. This will greatly facilitate understanding and discussion during the lecture. Students are encouraged to re-read the material after class for maximal value as the lecture will help to explain the reading. Incomplete slides will be available on the CANVAS course site (under Modules) so that students can review prior to lecture and use to facilitate note taking. Please also review the in-class assignment (under Modules) prior to class so that you do not need to take time to read the assignment during class. Web links to reading materials and original papers will be available on the course’s CANVAS site (Modules). Students are encouraged to ask all questions related to the course through CANVAS (Discussions). This system is highly catered to getting you help fast and efficiently from classmates and the instructor. Questions regarding personal issues should be emailed directly to the Course Director.

On the days that patient presentations are scheduled please understand that the patient is volunteering their time and often sharing deeply personal issues with the class. Remember that everything they say and their identity is strictly confidential. Do not discuss this with anyone outside of the class. Patient presentations will begin with me interviewing the patient to facilitate them telling their story. There will then be time for students to ask appropriate questions about the patient experience.

Course requirements and grading:

Attendance will be taken through an card swiper in the classroom. Please bring your Mcard daily to mark your attendance. Do not email the instructor to say you will be absent. An absence will be excused for compelling medical and other reasons where a letter from a reputable source is uploaded to Assignments within 48 hours of the due date. If you have COVID please upload documentation of a positive test result or a photo of a positive home test. In-class activities should only be done in-class. If you miss a class please do not upload the in-class assignment although you should do the activity on your own.

Late assignments and tests will not be accepted and a grade of 0 will be entered. An exception will be made for compelling medical and other reasons where a letter from a reputable source is uploaded to Assignments within 48 hours of the due date.

Final grades will be based on the following numerical system: highest grade-A+; 93 and above-A; 90-92-A-; 87-89-B+; 83-86-B; 80-82-B-; 77-79-C+; 73-76-C; 70-72-C-; 65-69-D; <65-F. I do not round up.

To pass the course all students are required to complete the Program for Education and Evaluation in Responsible Research and Scholarship (PEERRS) module on Human Subjects Research Protections, and upload the certificate of completion to Assignments in CANVAS by the due date. This is expected to take 1- 1 ½ hours. The link is… If you have already done this in the past and have a currently valid (not expired) certificate, you just need to upload that, you do not need to repeat the module.

Grading:

Mid-term 15%--multiple choice and short answer covering all lecture and readings up to the date of the mid-term. This exam will be given in class only. You will need a laptop or notebook that is capable of downloading a Word file from CANVAS, completing the exam and emailing/uploading it again when done. Students will have from 11:00 am- 12:20 to complete the exam.  The exam is completely closed-book. The exam will be available in Modules on this CANVAS site at 11:00 am. Please download the exam and change the filename to your ID number. So, if your ID number is 1234567 then the file would be 1234567.docx. Please only put your ID number on the exam. When done please email the exam to me and upload the exam to assignments in CANVAS. Since this is a closed book exam the only thing on your computer that you should view is the exam and the simple 4 function calculator. If you have a question during the exam please email it to me. If I have made a mistake on the exam or a question needs clarification I will make a verbal announcement to the entire class. If you need to leave to use the restroom or have a drink of water you may do so.

Cumulative Final 25%--multiple choice, short answer and short essay covering the whole course. The same in-class electronic type of exam as for the mid-term will occur. Please see mid-term above.

Class participation 10%--based on attendance and class participation in both lecture and the class activity parts of the class. Please note that unexcused absences from classes where we have patient presentations and classes 23-24, 26-28 will EACH reduce your class participation grade by 5%.

Paper 15%--a 4-5 page, Arial 12-point, double-spaced, 1 inch margin succinct and powerful essay. See the end of the syllabus for more specific information about the Paper.

Debates 20%--A group activity. Students will be asked to defend contrasting views on the role of evidence-based medicine in the care of patients. See the end of the syllabus for more specific information about the Debates.

Movie 15%--Students will prepare a 3 minute film interview with a person who has a story to tell about a good or bad interaction with the health care system. See the end of the syllabus for more description. All films will be shown together in class.

Academic Misconduct

The University community functions best when its members treat one another with honesty, fairness, respect, and trust. The College promotes the assumption of personal responsibility and integrity, and prohibits all forms of academic dishonesty and misconduct. All cases of academic misconduct will be referred to the appropriate office at the school of attendance. Being found responsible for academic misconduct will usually result in a grade sanction, in addition to any sanction from the College.

Diversity, Equity and Inclusion

The University School of Public Health seeks to create and disseminate knowledge, with the aim of preventing disease and promoting the health of populations worldwide. We recognize the histories of social discrimination globally and seek to promote and extend opportunities for members of all groups that historically have been marginalized. We commit to developing the institutional mechanisms and norms necessary to promote the values of diversity, equity, and inclusion, both inside and outside our classrooms. To this end, SPH upholds the expectations that all courses will (1) be inclusive, (2) promote brave discussions, (3) follow multicultural ground rules, and (4) abide by policies and procedures.

**Student Resources**

**Writing Lab:**

**Accommodations:**

**Student Wellness:**

**Support for Food Insecurity**:

**Sexual and Gender/race Harassment**:

**Course Schedule and List of Readings**

**Introduction**

1. 8/30—Lecture: Course introduction, syllabus review, nomenclature, examples of eminence and evidence-based medicine.

Class Activity: What do we *really* know about COVID-19?

Reading: Syllabus

2. 9/1--Lecture: Evolution and history of evidence-based medicine (EBM)

Class Activity: Patient presentation

Reading: 1. Text: Chapter 8: An Overview of Evidence-based medicine. 2. Evidence-based Medicine Working Group. Evidence-based medicine. A new approach to teaching the practice of medicine. *JAMA* 1992;268:2420-2425.

**Study Designs**

3. 9/6--Lecture: Identifying the problem: case reports and case series

Class activity: 1. Does measles vaccine cause injury? 2.

Reading: 1. Text: Chapter 5: Observational studies. 2. Putora PM et al. Swarm-based medicine. *Med Internet Res*. 2013 Sep 19;15(9):e207. Available at <http://www.jmir.org/2013/9/e207/> 3. Pneumocystis Pneumonia --- Los Angeles. MMWR June 5, 1981 / 30(21);1-3 available at <http://www.cdc.gov/mmwr/preview/mmwrhtml/june_5.htm>

4. 9/8--Lecture: Ecological studies

Class activity: 1. Is fast food bad for you? How to use PubMed to search (Emily Capellari)

Reading: 1. Text: Chapter 1: Epidemiology: Defining disease and normality. 2. Barr B et al. Impact on health inequalities of rising prosperity in England 1998-2007, and implications for performance incentives: longitudinal ecological study.
BMJ. 2012 Dec 4;345:e7831 3. Morgenstern LB et al. Fast food and neighborhood stroke risk. [*Ann Neurol.*](http://www.ncbi.nlm.nih.gov/pubmed/?term=fast+foodmorgenstern+lb) 2009 Aug;66(2):165-70.

5. 9/13--Lecture: Case control studies

Class activity: Discussion about the Death of Ivan Ilyich

Reading: 1. Text: Chapter 2: Measuring and summarizing data. 2. The Death of Ivan Ilyich by Leo Tolstoy.

6. 9/15--Lecture: Cohort studies

Class activity: Critique of Chan et al article

Reading: 1. Text: Chapter 3: Epidemiological concepts. 2. Chan M.T.V. et al. Association of Unrecognized Obstructive Sleep Apnea With Postoperative Cardiovascular Events in Patients Undergoing Major Noncardiac Surgery. *JAMA* 2019;321(18):1788-1798.

7. 9/20—Lecture: Clinical Trials I: Proposing the idea, protocol development, randomization, control, blinding, type I and II error

Class activity: Michicillin for the common cold

Reading: 1. Text: Chapter 4: Statistical inference, confidence intervals and p-values. 2. Text: Chapter 7: Investigating causes of disease (through P. 56 only). 3. Text: Chapter 11: Effectiveness.

8. 9/22--Lecture: Clinical Trials II: p values, systematic bias, effects and how to read a medical journal article.

Class activity: Michicillin for the common cold part II.

Reading: 1. Text: Chapter 9 (thru top of page 77 only): Diagnosis. 2. Chapter 10: Prognosis. 3. Echt DS, et al. Mortality and morbidity in patients receiving encainide, flecainide, or placebo. The Cardiac Arrhythmia Suppression Trial. New Eng J Med 1991;324:781-8.

**Ethics, law and communication**

9. 9/27— Guest lecture, Shared Decision Making and communicating with patients

Class Activity: Decision Aid construction

Reading: 1. [Elwyn G, et al. Developing a quality criteria framework for patient decision aids: online international Delphi consensus process. BMJ. 2006 Aug 26;333(7565):417](https://umich.instructure.com/courses/321636/files/11518648/download?wrap=1)

10. 9/29--Lecture: Evidence synthesis

Class activity: Patient presentation

Reading: 1. Text: Chapter 12: Systematic reviews and meta analysis. 2. Ding M et al. Long-Term Coffee Consumption and Risk of Cardiovascular Disease: A Systematic Review and a Dose-Response Meta-Analysis of Prospective Cohort Studies. *Circulation*. 2014;129:643-659.

11. 10/4— Guest Lecture, Regulatory law, the FDA and research misconduct

Class activity: Debate Introduction

Reading: 1. Research integrity.

1. <http://grants.nih.gov/grants/research_integrity/research_misconduct.htm> 2. Maher B. Sabotage. Nature. 2010;467:516-518.

**PAPER DUE BEFORE CLASS**

12. 10/6—Lecture: Human subject protection and the ethics of human research

Class activity: Mock IRB

Reading: 1. Text: Chapter 14: Audit, research ethics and research governance. 2. Rockwell DH et al. The Tuskegee study of untreated syphilis; the 30th year of observation. Arch Intern Med. 1964 Dec;114:792-8.

**PEERS Certificate Uploaded to CANVAS before class.**

13. 10/11--Lecture: Review for exam

Class activity: Guest:

Reading: None

**Health Disparities**

14. 14. 10/13--Lecture: Health Disparities

Class Activity: interview video and discussion

Reading: 1. Text: Chapter 18: Inequalities in health. 2. Text: Chapter 22: Global health. 3. Unequal treatment (Summary). <https://www.nap.edu/read/10260/chapter/2#3>

10/18—Fall break

15. 10/20--Midterm Exam

**The Art and Science of Medicine**

16. 10/25--Lecture: The art of medicine

Class activity:

Reading: 1. Healy B. Medicine, the Art. <http://www.cbsnews.com/news/medicine-the-art/>.

17. 10/27— Lecture: Religion, science and medicine

Class activity: 2. Debate preparation

Reading: 1. Campbell MK et al. Church-based health promotion interventions: evidence and lessons learned. Annu Rev Public Health. 2007;28:213-34. 2. Morgenstern LB et al. Fatalism, optimism, spirituality, depressive symptoms, and stroke outcome: a population-based analysis. Stroke. 2011 Dec;42(12):3518-23.

18.11/1--Guest Lecture:

Reading: Mom at Bedside, Appears Calm available at <http://www.nejm.org/doi/full/10.1056/NEJMp1312171#t=article>.

Class activity:. 2. Debate preparation

19. 11/3--Guest Lecture: Pre-clinical and animal studies

Class activity: Debate Preparation

Reading: 1. Kehinde EO. They See a Rat, We Seek a Cure for Diseases: The Current Status of Animal Experimentation in Medical Practice. Med Princ Pract. 2013 Nov 8:52-61.

20. 11/8— Guest Lecture: The apprentice model of training.

Class activity: Is Grey’s Anatomy Real?

Reading: 1. Kotsis SV, et al. Application of the "see one, do one, teach one" concept in surgical training. *Plast Reconstr Surg*. 2013 May;131(5):1194-201. 2. Stalmeijer RE et al. Clinical teaching based on principles of cognitive apprenticeship: views of experienced clinical teachers. *Acad Med*. 2013 Jun;88(6):861-5. 3. Gould DA et al. Simulation: moving from technology challenge to human factors success. *Cardiovasc Intervent Radiol*. 2012 Jun;35(3):445-53.

21. 11/10—Guest Discussant: How to care for "the difficult patient," and patients who "make bad decisions"

Lecture: Why is evidence-based medicine so threatening?

Reading: 1. 1.Saddawi-Konefka D, Schumacher DJ, Baker KH, et al. Changing Physician Behavior With Implementation Intentions: Closing the Gap Between Intentions and Actions. Acad Med. 2016 Sep;91(9):1211-6. 2. Salloch S. Who’s afraid of EBM? Medical professionalism from the perspective of evidence-based medicine. Med Health Care and Philos (2017) 20:61–66.

22. 11/15— Lecture: Advocacy for medical innovation.

Class activity: Patient presentation

Reading: 1. Wright J. Only your calamity: the beginnings of activism by and for people with AIDS. Am J Public Health. 2013 Oct;103(10):1788-98. 2. Riter B. History of Breast Cancer Advocacy [https://crcfl.net/history-of-breast-cancer-advocacy/Links to an external site.](https://crcfl.net/history-of-breast-cancer-advocacy/) **Movies due before class**.

**Putting it all together**

23: 11/17— Debates I and II

24. 11/22—Debate III and IV

11/24—Thanksgiving Break

25. 11/29— Guest Lecture: Careers in the science of medicine

Class activity: Catch up.

Reading: Text: Chapter 19: Health Improvement

26: 12/1— MOVIES!

27. 12/6—Movies and stories from a career in medicine.

28. 12/8—Lecture: Movies and review and exam preparation

29. 12/19 1:30-3:30 pm --Final exam.

**This is the ONLY time the final will be given.**

**Paper due before class on 10/4/2022**

A 4-5 page (not including references, DO NOT EXCEED 5 PAGES of text), Arial 12-point, double-spaced, 1 inch margin succinct and powerful essay. The first paragraph of the paper should include a position (thesis) statement and the essay should defend that position with the available literature. The literature cited should be critiqued as we discussed in class. Is the evidence original or someone else’s opinion? Is there objective evidence to suggest a clear direction? What are the study designs of the available literature and what is their position on the evidence ladder? Does the literature contain biases? What are they? All statements should be cited with references from the literature. Use of primary sources (journal articles) is strongly recommended. Avoid sentiment and stick to the facts. Focus on the clinical data and NOT on the basic science behind the drug. Please upload the paper to Assignments in CANVAS and email the paper to Professor (subject line should just be the digits from your UM ID number) prior to class on the due date. Please do NOT put your name on the paper; only put your ID number on the paper. I will download the papers into a file and randomly grade them.

The paper should have 10-20 references.

# Topic: Aducanumab (marketed as Aduhelm) received Accelerated Approval by the FDA to treat Alzheimer’s Disease. Based on data currently available should Aducanumab remain approved or should approval be withdrawn? Should insurance companies and Medicare pay for this treatment? Should physicians prescribe this medication for their patients.

Grading (15% of course grade) will include content, essay structure, communication skill, grammar, punctuation and spelling.

Rubric:

Content

Thesis statement (10)

Background (10)

Literature review and critique (30)

Evidence-based conclusion (20)

Style

Essay structure (10)

Convincing argument (10)

Grammar, punctuation, spelling (10)

**Debates**

Each debate will be 38 minutes. There will be two teams for each debate and they will randomly be assigned to take a pro or con position on a topical issue related to the science of medicine. The format will be 8 minute introductions where each team states their thesis with supportive evidence derived from our class discussions, readings and the available literature from searches. Please use original sources rather than media reports as much as possible. It is recommended that each group briefly discuss the disease in question (if relevant), state their position, and then **provide an evidence-base** to support their position. After both groups have given their introduction, a 10 minute interactive rebuttal will follow. In the remaining 12 minutes, 7 minutes will be used for questions from those not in the debate and 5 minutes for feedback to the debaters. Each member of the debating team must speak at one point, but it is acceptable to have one or more members do the majority of the talking if others have done the research or prepared the speaker. Everyone should contribute equally on each team. The topics listed below are hypothetical.

Grading (20% of the course grade). Grading will include preparation, knowledge, use of resources, communication skill, debating style, and, most importantly, participation as a team member in the debate. Those not currently debating will be asked to upload to CANVAS a one paragraph evaluation of each debate pointing out the strengths and weaknesses of each side. Please upload this on the last day of the debates.

Please note that these debate topics are hypothetical.

Topic 1: Should mask mandates be imposed when the community risk level for COVID-19 is high? The Pro Team will argue yes and the Con team no.

Topic 2: The U.S. spends a modest amount of money on global health. Should that amount be greatly increased. The Pro Team will argue yes and the Con Team no.

Topic 3: Medical science has become politicized. Should there be an independent branch of government to determine research funding, drug and device approval, and have regulatory authority to mandate health behavior across the U.S? The Pro Team will argue yes and the Con team no.

Topic 4: Tele-health grew tremendously during the pandemic. Post-pandemic, many insurers plan to stop paying for tele-health services. Should tele-health continue to be funded by Public and Private insurers? The Pro Team will argue yes and the Con Team no.

Rubric:

Content:

Framing of the issue: 10 points

Using original sources to support argument: 20 points

An evidence-based approach to argument: 20 points

Rebuttal:

Being prepared and effective rebuttal: 20 points

Teamwork: 30 points**Movie**

Students will prepare a 3 minute (not more than 3 minutes!) movie using either iMovie (greatly preferred) or Windows Movie Maker. Students must get anyone who appears on camera to sign a release. A blank copy is available in the class CANVAS site. Completed forms must be uploaded to Assignments to get credit for the assignment. The video will begin with the camera on the student who will introduce themselves. The student will then film the student interacting with the subject **but will not identify the subject by name**. Only one story may be included in the film but more than one person can be in the film (eg. Spouse, parent, etc). The student will interview the subject and the focus will be on the subject telling a story about a healthcare interaction that illustrates the good, the bad, and/or the ugly of that interaction. **The story should be instructive** and point out lessons from the class, eg. shared decision making, the art of medicine, special populations, the physician sharing evidence, etc. **How did the health care professional use (or not use) evidence to guide the decision-making process?** The subject of the interview needs to provide factual information rather than descriptors. A person saying “I really hated that doctor,” is not informative. What did the doctor do that was bad and what would have made it better? How did the person grow or learn from the experience? How does the story reflect evidence-based medicine? How has it changed the subject’s approach to healthcare? Make sure you help your subject understand what scientific data means. A doctor saying “I’ve done this a million times and all my patients do well” is not providing scientific data. You must ask the question if the doctor provided data from published research studies or just their own personal experience. Students will be graded on the quality of the film including content and whether it brings out points from the class (especially evidence-based practice), originality, cinematography and design. The stories should be truthful, not made up stories (I can tell!) Please upload your video to Dropbox using the link (to be provided). Please name your file with your unique name and appropriate file extension, so if your unique name is jgj than your file should be jgj.mov. Acceptable file extensions are .mov (preferred) or .mp4.

Rubric:

Content: does the story provide useful material supporting class material: 35 points

Framing: does the student set the stage and put it together in the end: 25 points

Cinematography: is the video easy to see and sound easy to hear: 20 points

Originality: is the story original and believable: 20 points
